# Nasogastric- vs. percutaneous gastrostomy tube for prophylactic gastric decompression after cytoreductive surgery with hyperthermic intraperitoneal chemotherapy

**DOI:** 10.1515/pp-2021-0107

**Published:** 2021-03-24

**Authors:** Job P. van Kooten, Nadine L. de Boer, Marjolein Diepeveen, Cornelis Verhoef, Jacobus W. A. Burger, Alexandra R. M. Brandt-Kerkhof, Eva V. E. Madsen

**Affiliations:** Department of Surgical Oncology and Gastrointestinal Surgery, Erasmus MC Cancer Institute, Rotterdam, The Netherlands; Department of Surgery, Catharina Hospital Cancer Institute, Eindhoven, The Netherlands

**Keywords:** cytoreductive surgery (CRS), gastric decompression, hyperthermic intraperitoneal chemotherapy (HIPEC), length of stay (LOS), nasogastric tube, percutaneous gastrostomy, postoperative outcomes

## Abstract

**Objectives:**

Cytoreductive surgery (CRS) with hyperthermic intraperitoneal chemotherapy (HIPEC) is associated with postoperative gastroparesis and ileus. In 2015, our practice shifted from using percutaneous gastrostomy tubes (PGT), to nasogastric tubes (NGT) for prophylactic gastric decompression after CRS-HIPEC. This study aimed to compare these methods for length of stay (LOS) and associated complications.

**Methods:**

Patients that underwent CRS-HIPEC for peritoneal metastases from colorectal cancer between 2014 and 2019 were included. Cases were grouped based on receiving NGT or PGT postoperatively. Multivariable linear regression determined the independent effect of decompression method on LOS, thereby adjusting for confounders.

**Results:**

In total, 179 patients were included in the analyses. Median age was 64 years [IQR:54–71]. Altogether, 135 (75.4%) received a NGT and 44 (24.6%) received a PGT. Gastroparesis occurred significantly more often in the PGT group (18.2 vs. 7.4%, p=0.039). Median LOS was significantly shorter for patients with a NGT (15 [IQR:12–19] vs. 18.5 [IQR:17–25.5], p<0.001). PGT was independently associated with longer LOS in multivariable analysis (Beta=4.224 [95%CI 1.243–7.204]). There was no difference regarding aspiration, pneumonia and postoperative mortality between groups.

**Conclusions:**

NGT should be preferred over PGT for gastric decompression after CRS-HIPEC as it is associated with fewer gastroparesis and shorter LOS.

## Introduction

Colorectal carcinoma (CRC) is one of the most prevalent forms of cancer. Approximately 8% of patients will at some point develop peritoneal metastases (PM) [1], [2], [3]. A potentially curative treatment for PM is cytoreductive surgery with hyperthermic intraperitoneal chemotherapy (CRS-HIPEC) [4], [5]. This procedure is associated with considerable postoperative morbidity [6]. Postoperative nausea and delayed gastro-intestinal recovery often occur. Also patients suffer from postoperative ileus more frequently compared to patients undergoing conventional colorectal cancer surgery [7], [8], [9]. Currently there is no consensus on the routine use of gastric decompression, nutritional supplementation, or ‘early recovery after surgery’ (ERAS) practices after CRS-HIPEC. A recent survey by Maciver et al. among 97 high-volume CRS-HIPEC surgeons revealed that 74% does not apply ERAS protocols, 83% routinely places nasogastric tubes (NGT) and 59% routinely uses nutritional supplementation of which 18% consists of enteral feeding through NGTs or percutaneous gastrostomy tubes (PGT) [10]. At the authors’ hospital, gastric decompression is routinely performed. At the start of the CRS-HIPEC program at this hospital, this was achieved via a PGT placed during surgery. Possible advantages of a PGT over NGT are less dislocation of the tube, less respiratory tract infections and especially less patient discomfort [11]. NGT on the other hand is considered less invasive, causes fewer wound infections and can be removed and replaced early postoperatively thereby possibly decreasing postoperative length of stay (LOS) [12], [13], [14]. Therefore a NGT was used for gastric decompression from 2015 onwards at this center. Though Maciver showed that its use is probably limited, some centers apply PGTs for gastric decompression and enteral feeding. The aim of this study was to identify a preferable practice by comparing both gastric decompression methods in terms of associated postoperative complications, and length of stay (LOS) in hospital.

## Materials and methods

### Patient selection

All patients undergoing CRS-HIPEC for PM from CRC between March 2014 and February 2019 in the Erasmus MC Cancer Institute were identified from a prospective database. Cases not undergoing full CRS-HIPEC (i.e. open-close procedures, debulking only or HIPEC only) were excluded from statistical analyses. This study was approved by the local Medical Ethics Review Committee (MEC-2018-1286).

### CRS-HIPEC procedure

CRS-HIPEC procedures were performed according to the Dutch CRS-HIPEC protocol [15]. In short: Peritoneal cancer index (PCI) according to Sugarbaker was determined after median laparotomy [16]. For most patients PCI was also determined several weeks up front via diagnostic laparoscopy. Only patients with PCI below or equal to 20 points were amenable for CRS-HIPEC. HIPEC was administered with the open coliseum technique. Regimens used were Mytomycin-C (MMC) (35 mg/m^2^) at 41–42 °C for 90 min or Oxaliplatin (460 mg/m^2^) in combination with systemic folinic acid (20 mg/m^2^) and 5-fluorouracil (5-FU; 400 mg/m^2^) added to iso-osmotic dialysis solution (Dianeal^®^) at 41–42°C for 30 min. When necessary, bowel anastomosis and/or stomy procedures were performed after HIPEC perfusion. Prophylactic gastric decompression was achieved by either placing a PGT or NGT intraoperatively, together with an enteral feeding tube via the same route.

### Postoperative course

All patients were admitted to the intensive care unit (ICU) postoperatively, according to local protocol. After sufficient stabilization, patients were transferred to the surgical oncology ward. According to the Erasmus MC Cancer Institute postoperative CRS-HIPEC protocol, all gastric decompression tubes had to stay opened for at least three days and were then clamped when they produced less than 1000 mL per 24 h. During the first three postoperative days, patients had no oral intake and nutritional support was provided by enteral feeding for at least four days. After day three, enteral nutritional support was abated based on the recovery of oral intake from day four onwards. Following clamping of the gastric decompression tube, gastric retention was observed during the day. The tube was removed if gastric retention was less than 250 mL per day. In case of PGT, tubes could only be removed after a minimum of 10 days to secure the forming of a gastro-cutaneous fistula. Prophylactic PGT placement was routinely performed until the end of 2015. After 2015 the protocol for postoperative care for CRS-HIPEC patients changed, after which it was standard practice to use a NGT for prophylactic gastric decompression.

### Complications

All postoperative complications were graded following the classification as described by Clavien and Dindo [17], [18]. In short: grade I includes any deviation from normal postoperative course; grade II comprises complications requiring pharmacological treatment; grade IIIa comprises complications requiring surgical, endoscopic or radiological intervention not under general anesthesia; grade IIIb comprises complications requiring surgical, endoscopic or radiological intervention under general anesthesia; grade IV comprises life threatening complications requiring ICU management in which grade IVa includes single organ dysfunction and IVb includes multi organ dysfunction; grade V comprises post-operative mortality. For patients with multiple complications, the highest grade complication was registered.

### Statistical analysis

**Figure 1:**
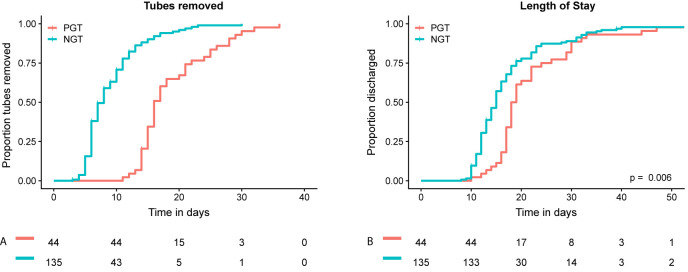
Cumulative incidence analyses for tube removal and hospital discharge. Cumulative proportion of gastric decompression tubes removed after CRS-HIPEC (A). Cumulative proportion of discharged patients (B). NGT=nasogastric tube, PGT=percutaneous gastrostomy tube.

**Figure 2:**
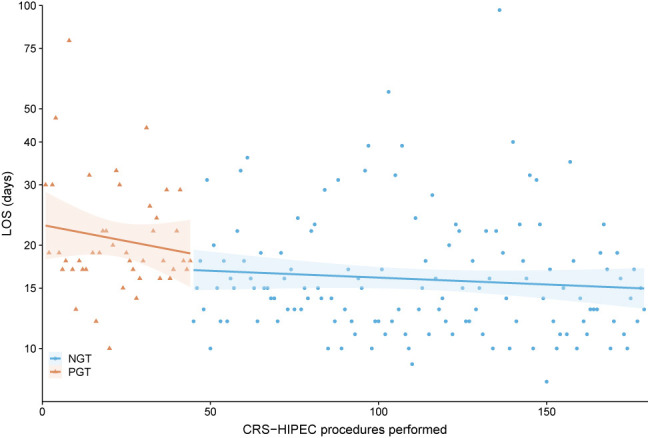
Length of stay (LOS) per gastric decompression method (dots and triangles), ranked in time with linear regression estimate of LOS (line) and 95% confidence interval of estimate (transparent area). A direct drop in LOS was observed after switching from PGT to NGT for prophylactic gastric decompression. PGT (triangles)=percutaneous gastrostomy tube. NGT (dots)=nasogastric tube.

Continuous variables were presented as median with interquartile range (IQR). Categorical variables were presented as absolute numbers with percentages. Cases were grouped based on type of gastric decompression tube: NGT vs. PGT. Length of stay (LOS) was chosen as main outcome, as this was considered to be an important and objective measure of postoperative outcome, available for all patients. Baseline characteristics, intraoperative and postoperative course between both groups were compared, to be able to adjust for variables associated with LOS and to assess complications that could be associated with gastric decompression method, such as gastroparesis and pneumonia. Continuous variables were compared using the Mann-Whitney U test. Pearson’s Chi-squared test was used to compare proportions. Fisher’s exact test was applied when less than five events occurred in a group. Two sided p-values <0.05 were considered statistically significant. To determine the effect of gastric decompression method on LOS, corrected for other variables that impact LOS, multivariable linear regression with backward selection was used. Variables, known to impact LOS or are associated with surgeon experience, were entered in the model: age, gender, ASA score, BMI, PCI, duration of surgery, blood loss during surgery, gastric decompression method (i.e. NGT vs. PGT) and postoperative complications (any grade). Statistical analyses were performed using Statistical Package for Social Sciences (SPSS) version 25.0.0.1 (IBM Corporation, Armonk, NY, USA). Cumulative incidence curves were generated, to visualize the duration of tube placement and LOS for both groups (Figure 1A and B).To visualize the course of LOS over time, thereby comparing both gastric decompression methods, LOS was plotted for each individual patient and a linear regression estimate was fitted to the data (Figure 2). All figures were created using R version 3.5.1 (http://www.r-project.org).

## Results

### Patient selection

During the study period 235 CRS-HIPEC procedures for PM from CRC were performed. Open-close (n=34, 14.5%), HIPEC only (n=17, 7.2%) and CRS/debulking only procedures (n=5, 2.1%) were excluded from analyses. In total 179 cases (76.2%) were included for statistical analyses, of which 135 (75.4%) received NGT and 44 (24.6%) PGT.

### Baseline characteristics

At baseline, median BMI was significantly higher in the NGT group (26.6 [IQR 23.4–30.1] vs. 24.4 [IQR 22.3–26.5]) (Table 1). Other baseline characteristics (age, sex, smoking status, ASA-classification, primary tumor location and time of PM diagnosis) did not differ significantly between patients receiving NGT vs. PGT.

**Table 1:** Baseline characteristics.

	Total	NGT	PGT	p-Value
	n=179	n=135	n=44	
Gender				
Male	91 (50.8)	74 (54.8)	17 (38.6)	0.062
Female	88 (49.2)	61 (45.2)	27 (61.4)	
Age, years	64 [54–71]	65 [54–72]	61.5 [51.5–68]	0.204
BMI, kg/m^2^	26.1 [22.9–29.2]	26.6 [23.4–30.1]	24.4 [22.3–26.5]	0.017
Smoking (past or current)				
Yes	90 (50.3)	70 (51.9)	20 (45.5)	0.526
No	82 (45.8)	59 (43.7)	23 (52.3)	
Unknown	7 (3.9)	6 (4.5)	1 (2.3)	
ASA-classification				
1	28 (15.7)	18 (13.4)	10 (22.7)	0.437
2	117 (65.7)	90 (67.2)	27 (61.4)	
3	31 (17.4)	24 (17.9)	7 (15.9)	
Unknown	2(1.1)	2 (1.5)	0 (0)	
Primary tumor location				
Appendix	2 (1.1)	2 (1.5)	0 (0)	0.607
Ascending colon	68 (38)	48 (35.6)	20 (45.5)	
Transverse colon	12 (6.7)	9 (6.7)	23 (6.8)	
Descending colon	17 (9.5)	15 (11.1)	2 (4.5)	
Sigmoid	46 (25.7)	33 (24.4)	13 (29.5)	
Rectum	25 (14)	20 (29.5)	5 (11.4)	
Unknown	9 (5)	8 (5.9)	1 (2.3)	
PM diagnosis				
Synchronous	77 (43)	57 (42.2)	20 (45.5)	0.707
Metachronous	102 (57)	78 (57.8)	24 (54.5)	

### Surgical procedure

To assess whether LOS could have been influenced by differences in intra-operative course, the surgical procedures for both groups were compared. During surgery the NGT group suffered less blood loss (0.9 [IQR 0.5–1.4] vs. 1.5 [IQR 0.9–2.8] liters, p<0.001) and the median duration of the operative procedure was shorter for this group (346 [IQR 292–410] vs. 428 [IQR 400–508] minutes, p<0.001) (Table 2). The number of resections was comparable between groups, with the exception of the number of pelvic organ resections. In the PGT group, significantly more pelvic organ resections were performed (54.5 vs. 34.8%, p=0.020). This difference was caused in particular by the number of hysterectomies, which was 36.4% (n=16) for the PGT group vs. 13.3% (n=18) for the NGT group (p=0.001).

**Table 2:** Intra-operative characteristics.

	Total	NGT	PGT	p-Value
	n=179	n=135	n=44	
PCI	11 [6–16]	10 [6–15]	12 [6–16]	0.147
R-score				
R1	176 (98.3)	133 (98.5)	43 (97.7)	0.599
R2a	1 (0.6)	1 (0.7)	0 (0)	
R2b	2 (1.1)	1 (0.7)	1 (2.3)	
Procedure time, min	382 [311–439]	346 [292–410]	428 [400–508]	**<0.001**
Blood loss, L	1.0 [0.6–1.6]	0.9 [0.5–1.4]	1.5 [0.9–2.8]	**<0.001**
HIPEC regimen				
MMC	159 (88.8)	117 (86.7)	42 (95.5)	0.166
Oxaliplatin	20 (11.2)	18 (13.3)	2 (4.5)	
Resections				
Omentectomy	171 (95.5)	128 (94.8)	43 (97.7)	0.417
Peritonectomy (≥1)	131 (73.2)	100 (74.1)	31 (70.5)	0.638
Diaphragm	31 (17.3)	22 (16.3)	9 (20.5)	0.527
Gastrectomy	2 (1.1)	2 (1.5)	0 (0)	1.000
Small bowel	53 (29.6)	38 (28.1)	15 (34.1)	0.453
Colon/rectum	110 (61.5)	81 (60)	29 (65.9)	0.484
HPB (including Splenectomy)	27 (15.1)	18 (13.3)	9 (20.5)	0.252
Pelvic organs^a^	71 (39.7)	47 (34.8)	24 (54.5)	**0.020**
Anastomosis				
Yes	96 (53.6)	66 (48.9)	30 (68.2)	**0.026**
Median number/patient	1 [1–1.25]	1 [1–1]	1 [1–2]	0.639
Stomy				
Ileostomy	9 (11.8)	4 (7.4)	5 (22.7)	0.111
Colostomy	67 (37.4)	50 (37)	17 (38.6)	0.849

^a^Pelvic organs comprises urinary bladder, ovaries, uterus and ureters. p-Values in bold are statistically significant.

### General postoperative course

Postoperative course did differ considerably between both groups (Table 3). Median duration of PGT placement was longer compared to NGT (16 days [IQR 15–21] vs. 7 days [IQR 6–10] (Figure 1A). Patients that had a PGT suffered from gastroparesis significantly more often (18.2 vs. 7.4%, p=0.039). Complications (any grade) occurred more often in the PGT group (72.7 vs. 54.1%, p=0.029). There was no significant difference in number of high grade complications (i.e. Clavien-Dindo grade 3 or higher) (25.2% for NGT vs. 18.2% for PGT, p=0.341) or re-operations (14.6% for NGT vs. 16.3% for PGT, p=0.795).

**Table 3:** Postoperative outcome.

	Total	NGT	PGT	p-Value
	n=179	n=135	n=44	
Duration of tube placement, days	9 [6–15]	7 [6–10]	16 [15–21.8]	
Length of stay, days	16 [13–20]	15 [12–19]	18.5 [17–25.5]	**<0.001**
Complications (any grade)	105 (58.7)	73 (54.1)	32 (72.7)	**0.029**
Complications Clavien-Dindo≥III	42 (23.5)	34 (25.2)	8 (18.2)	0.341
Reoperations	25 (15.1)	18 (14.6)	7 (16.3)	0.795
Clavien-Dindo grade				
I	24 (13.4)	14 (19.2)	10 (31.3)	0.399
II	39 (21.8)	25 (34.2)	14 (43.8)	
IIIa	16 (8.9)	13 (17.8)	3 (9.4)	
IIIb	19 (10.6)	14 (13.3)	5 (15.6)	
IVa	3 (1.7)	3 (4.1)	0 (0)	
IVb	1 (0.6)	1 (1.4)	0 (0)	
V	3 (1.7)	3 (2.9)	0 (0)	
Complications				
Gastroparesis	18 (10.1)	10 (7.4)	8 (18.2)	**0.039**
Aspiration	4 (2.2)	4 (3)	0 (0)	0.573
GI leakage^a^	11 (6.1)	9 (6.7)	2 (4.5)	0.611
Post-operative hemorrhage	7 (3.9)	4 (3)	3 (6.8)	0.252
Intra-abdominal abscess	18 (10.1)	14 (10.4)	4 (9.1)	0.806
POWI	16 (8.9)	13 (9.6)	3 (6.8)	0.570
Wound dehiscence	3 (1.7)	2 (1.5)	1 (2.3)	0.723
Ileus	6 (3.4)	6 (4.4)	0 (0)	0.155
Pulmonary embolism	3 (1.7)	2 (1.5)	1 (2.3)	0.723
Pneumonia	8 (4.5)	8 (5.9)	0 (0)	0.099
Cardiac complications	7 (3.9)	5 (3.7)	2 (4.5)	0.802
UTI	18 (10.1)	12 (8.9)	6 (13.6)	0.363

^a^GI leakage comprises anastomotic leakage and postoperative bowel ischemia or perforation. p-Values in bold are statistically significant.

### Pneumonia

In the NGT group eight patients developed pneumonia, against no patients in the PGT group. This difference was however not significant (Fishers exact test p=0.12). Because pneumonia could potentially be caused by aspiration, all cases were reviewed. There was no link between pneumonia and aspiration in six cases. In one case, aspiration was not likely but could not be ruled out definitely. In one case, aspiration had caused the pneumonia.

### Postoperative mortality

There was no significant difference in postoperative mortality between groups. There were five cases (2.8%) in the NGT group vs. no cases in the PGT group. Three of these patients died of multi organ failure from sepsis caused by anastomotic leakage. Two patients died because of respiratory failure caused by aspiration. In one of these patients the NGT was removed five days before this incident. In the other patient, the NGT was still in place.

### Length of stay

Median LOS for the entire cohort was 16 days (IQR 13–20) (Table 3). Median LOS was significantly longer for patients with a PGT (18.5 days (IQR 17–25.5) vs. 15 days (IQR 12–19), p=<0.001) (Figure 1B). Figure 2 shows the immediate decrease in LOS after postoperative management changed, replacing PGT for NGT. Multivariable regression analysis showed that gastric decompression method (Beta for PGT=4.224 [95% CI 1.243–7.204], p=0.006) and occurrence of postoperative complications from any grade (Beta 7.422 [95% CI 4.691–10.154]) were independently associated with LOS (Table 4).

**Table 4:** Linear regression analysis for length of stay (LOS) after CRS-HIPEC.

	Univariate	p-Value	Multivariable	p-Value
	Beta [95% CI]		Beta [95% CI]	
Age, years	−0.017 [−0.163–0.129]	0.819		NS
Gender				
Male	1			
Female	−1.046 [−4.291–2.199]	0.526		NS
ASA score				
ASA 1	1			
ASA 2	2.081 [−2.475–6.36]	0.369		NS
ASA 3	3.007 [−2.664–8.678]	0.297		NS
BMI, kg/m^2^	0.171 [−0.147–0.489]	0.289		NS
PCI	0.116 [−0.168–0.400]	0.423		
Blood loss, L	2.198 [0.973–4.022]	0.001		NS
Duration of surgery, minutes	0.012 [−0.003–0.027]	0.120		NS
Postoperative complications (any grade)	8.266 [5.210–11.321]	<0.001	7.422 [4.691–10.154]	<0.001
Gastric decompression method				
NGT	1		1	
PGT	5.076 [1.417–8.735]	0.007	4.224 [1.243–7.204]	0.006

## Discussion

This study shows that PGT for prophylactic gastric decompression and enteral feeding after CRS-HIPEC results in prolonged LOS when compared to NGT. Also, PGT appears to result in more cases of gastroparesis. Therefore NGT should be preferred over PGT for prophylactic gastric decompression after CRS-HIPEC.

### Routine gastric decompression after CRS-HIPEC

The prophylactic use of gastric decompression after CRS-HIPEC has long been debated, and early recovery after surgery (ERAS) protocols have not been widely adopted for CRS-HIPEC as of yet [12], [13], [19]. Due to the extent of CRS-HIPEC procedures, often involving omentectomy, peritonectomy and multiple organ resections, there is a considerable amount of patients suffering from postoperative intestinal paralysis (i.e. gastroparesis and/or ileus) [4], [7], [20], [21]. Patients often also receive opioids for postoperative pain management, increasing the risk for prolonged intestinal paralysis and pulmonary aspiration. Arakelian et al. showed that oral intake is restored after a median of 10 days, and postoperative nausea persists for a median of 11 days in a cohort of patients undergoing CRS-HIPEC [8]. Simkens et al. showed that the number of patients suffering from postoperative ileus is almost three times higher after CRS-HIPEC compared to conventional colorectal surgery [7]. A prospective study among patients undergoing CRS-HIPEC in the UK and Denmark revealed that over half of patients developed prolonged postoperative ileus [22]. The exact mechanism of action by which this is caused is not exactly clear. One hypothesis is that this is caused by the resection of the right gastro-epiploic artery (GEA) during omentectomy. Evers et al. conducted a randomized trial, preserving the right GEA in one group and resecting it in the other group. They found no association between resection or preservation of the right GEA and gastric emptying postoperatively. They suggested that the extensive intestinal manipulation or the heated intra-peritoneal chemotherapy were more probable causes [23]. Though the mechanism is not exactly clear, it remains a fact that patients suffer from gastro-intestinal paralysis more often after CRS-HIPEC compared to other colorectal or gastro-intestinal surgeries. This has caused surgeons to be reluctant with the abolition of routine gastric decompression after CRS-HIPEC. A 2017 survey by Maciver et al. revealed that 83% of CRS-HIPEC surgeons routinely place a NGT [10]. About three to five percent routinely use a PGT. The recently published guidelines for perioperative care in CRS-HIPEC state that prophylactic gastric decompression should not be performed at all, but this recommendation is based on indirect evidence and the recommendation strength is graded as ‘weak’ [24]. White et al. assessed the impact of the implementation of an ERAS protocol for patients undergoing CRS-HIPEC by comparing outcomes before and after implementation [25]. They found that ERAS practices are feasible for patients undergoing CRS-HIPEC. The mean LOS was shorter for the ERAS group and serious complications occurred less frequently. Though ‘not routinely using NGTs’ was part of their ERAS protocol, they did not report on the number of patients needing gastric decompression in the ERAS group. Nor did they report on complications associated with gastroparesis or ileus. Moreover the individual impact of not routinely using NGTs on their outcome measures cannot be determined. Thus, these studies and recommendation do not decisively establish that routine gastric decompression should be avoided, and as mentioned it is still widely applied. Also a majority of CRS-HIPEC surgeons apply routine nutritional supplementation, of which almost 20% is provided by enteral feeds through NGTs and PGTs. The aim of this study was not to defend the use of routine gastric decompression though, but rather to establish a data based rationale for a preferred route when routine decompression is applied.

### Impact of gastric decompression method on hospital stay

So, the majority of CRS-HIPEC surgeons use routine gastric decompression and a small proportion uses PGTs to achieve this. On the one hand, a PGT might be preferred as it less often dislocates and is considered to be less inconvenient by patients [11]. A major disadvantage of using a PGT is the fact that it is more invasive, it has to remain in situ for a longer period of time and is associated with an increased rate of infections compared to a NGT [14]. A NGT on the other hand can cause airway infections and is considered more inconvenient by patients [13]. As this study also shows however, it has the advantage that it can be removed earlier, possibly resulting in earlier restart of oral intake and fewer cases of prolonged gastroparesis. Moreover, in the current cohort the use of a NGT is associated with shorter LOS compared to PGT. In the protocol that was used, PGT had to remain in place for at least 10 days to secure the forming of a gastro-cutaneous fistula. This contributes to the fact that the median number of days that the gastric decompression tube was in place was much longer (16 days) for the PGT group compared to the NGT group (seven days). The duration of tube placement is difficult to compare due to the different protocols for both methods, requiring a 10 day minimum for the PGT group. However, this large difference between both methods does illustrate a clear disadvantage of the PGT. Moreover, the 10 day requirement for PGTs did not affect the main outcome of this study, i.e. LOS. As can be seen in Figure 1B, only two patients in the total cohort were discharged before day 10. This indicates that almost 99% of patients were still in hospital at day 10, regardless of gastric decompression method.

### Postoperative morbidity and mortality

An important observation in this cohort was that gastroparesis occurred significantly more frequently in the PGT group, suggesting that PGT results in gastroparesis more often than NGT. No additional analyses regarding the independent association between gastric decompression method and gastroparesis was performed due to small number of events. There was however no significant differences at baseline for possible risk factors associated with gastroparesis, such as age, gender, diabetes, number of gastrectomies or omentectomies. Prolonged gastroparesis delays postoperative recovery [26], [27]. And as gastroparesis is considered a risk factor for aspiration, it should absolutely be prevented [28], [29]. For this cohort, all cases of pneumonia were reviewed as this can be a clinical outcome of aspiration. Although all eight cases of pneumonia were in the NGT group, this difference was not significant. In six of eight cases there was no apparent link between the pneumonia and aspiration. In one case aspiration could not be ruled out by review of the patient chart. However, this patient was treated with regular antibiotics for hospital required pneumonia, and not with the adapted regimen for aspiration pneumonia. Therefore it seems unlikely that this pneumonia was caused by aspiration. In just one case, the pneumonia was definitely caused by aspiration. These outcomes imply there is no statistical difference for the occurrence of aspiration between both decompression methods.

At last all causes of postoperative death were reviewed. There was a non-significant difference, with all of five cases of postoperative death occurring in the NGT group. Three of these patients died of sepsis caused by anastomotic leakage. Two patients had to be resuscitated an eventually died due to massive aspiration. The NGT was removed five days before this event in one of these patients. In the other case, the NGT was still in place at time of aspiration. Though all cases of aspiration took place in the NGT group, this difference was not significant and the number of events was too small to draw valid conclusions regarding the risk of aspiration for different gastric decompression methods.

### Strengths and limitations

The main limitation of this study is the fact that PGT was only used in the beginning years of CRS-HIPEC at this center. Therefore the learning curve of surgeons performing the procedure could also have contributed to prolonged LOS in the NGT group, thereby biasing this outcome. Especially the intra-operative characteristics reflect the impact of surgeon experience, as operation time and blood loss are significantly higher for the PGT group. Also the percentage of postoperative complications is higher in the PGT group, which can possibly be explained by surgeon experience. Therefore, duration of surgery, perioperative blood loss and postoperative complications were added in the multivariable model, to correct for the possible association between surgeon experience and LOS. Nonetheless, gastric decompression method was independently associated with LOS. The most predictive variable for LOS was occurrence of postoperative complications. This indicates that surgeon experience, though it is associated with LOS, cannot account for the full difference between groups. Also, a direct decrease in LOS was seen after switching from PGT to NGT (Figure 2). If the difference in LOS between both groups was only caused by increasing surgeon experience and routine, one would expect a gradual decline in LOS rather than the direct decrease that was observed. Also it is not clear to what degree surgeon experience is associated with LOS in the current situation. Polanco et al. studied the learning curve for CRS-HIPEC in their hospital in Pittsburgh (PA, USA) and showed that LOS decreases with experience and reaches a plateau after approximately 90 procedures [30]. However, Kuijpers et al. evaluated the learning curve for three centers in the Netherlands and found that new centers that were trained by an experienced CRS-HIPEC center had non inferior postoperative morbidity rates [31]. LOS was also similar to that of the more experienced center. Surgeons in the Erasmus MC Cancer Institute were trained by the same surgeons and followed the same training program as the hospitals that were analyzed in their study. Our findings are similar to that of Kuijpers et al. suggesting the effect of learning curve on postoperative outcome is limited in this cohort. This is also confirmed by multivariable linear regression analysis, as mentioned before.

Another limitation is the retrospective nature of the study. But besides a significant difference in BMI between groups, baseline characteristics were comparable. BMI was not associated with LOS in univariate or multivariable analysis, thus this baseline difference did likely not affect the main outcome. The retrospective nature also limited the analyses, as there were little data available on the restart of oral intake, recovery in general and patient experienced inconvenience. If one would design a prospective study, these factors should be taken into account.

Regardless of these limitations, this study was able to show that prophylactic gastric decompression via a NGT should be preferred over PGT after CRS-HIPEC, as it is associated with shorter LOS, fewer gastropareses and is not associated with more postoperative complications. To our knowledge this is the first study looking in to this subject for CRS-HIPEC patients. Especially for this group of patients, that often suffers from (severe) complications and long term hospital stay, these kind of analyses are important to provide data and evidence for ‘best practice’ in postoperative care.

## Conclusions

PGT for gastric decompression results in prolonged LOS when compared to NGT after CRS-HIPEC. PGT also appears to result in more cases of gastroparesis. For prophylactic gastric decompression, a NGT should therefore be preferred over a PGT.
